# Peripheral Leukocyte Syndecan-3 Is Elevated in Alzheimer’s Disease: Evidence from a Human Study

**DOI:** 10.3390/ijms26146587

**Published:** 2025-07-09

**Authors:** Anett Hudák, Annamária Letoha, Tamás Letoha

**Affiliations:** 1 Pharmacoidea Ltd., H-6726 Szeged, Hungary; anett.hudak@pharmacoidea.eu; 2 Department of Internal Medicine, Albert Szent-Györgyi Clinical Center, Faculty of Medicine, University of Szeged, H-6720 Szeged, Hungary; letoha.annamaria@med.u-szeged.hu

**Keywords:** Alzheimer’s disease, syndecan-3, peripheral blood mononuclear cells, biomarker, p-tau217, neuroinflammation, immune remodeling

## Abstract

Syndecan-3 (SDC3), a transmembrane heparan sulfate proteoglycan involved in cell signaling and endocytosis, has recently been implicated in the pathogenesis of neurodegenerative disorders. While preclinical studies have demonstrated its role in Alzheimer’s disease (AD), its diagnostic relevance in peripheral blood remains unexplored. In this human cohort study, we measured SDC3 expression in peripheral blood mononuclear cells (PBMCs) from 22 clinically diagnosed AD patients and 20 cognitively unimpaired non-AD controls using a custom ELISA. The findings were compared with plasma p-tau217 levels and a panel of systemic laboratory markers. PBMC-expressed SDC3 was significantly elevated in AD patients and moderately correlated with AD status (*r* = 0.309, *p* = 0.0465) independent of age. Notably, SDC3 levels were inversely correlated with systemic inflammatory markers, including C-reactive protein (CRP; *r* = −0.421, *p* = 0.0055) and D-dimer (*r* = −0.343, *p* = 0.038), suggesting an AD-associated immune phenotype distinct from acute-phase or vascular inflammation. Conversely, plasma p-tau217 levels did not significantly differ between groups but correlated with markers of tissue injury and inflammation (LDH, GOT, and ferritin), potentially reflecting systemic influences in non-AD controls. A multivariable logistic regression model incorporating SDC3, p-tau217, and age demonstrated high diagnostic accuracy (AUC = 0.85). These findings identify PBMC-expressed SDC3 as a promising blood-based biomarker candidate for AD, warranting further validation in larger, biomarker-confirmed cohorts.

## 1. Introduction

Alzheimer’s disease (AD) is the most prevalent form of dementia globally, characterized by progressive cognitive decline, extracellular amyloid-beta (Aβ) plaque accumulation, intracellular neurofibrillary tangles composed of hyperphosphorylated tau, and sustained neuroinflammation [1,2]. Despite significant advances in elucidating AD pathogenesis, therapeutic breakthroughs remain limited [3]. The first disease-modifying therapies recently approved in the United States and Japan show clinical benefits only in the early stages of the disease [4]. However, current diagnostic tools such as cerebrospinal fluid (CSF) analysis and amyloid PET imaging are invasive, expensive, and not widely available [5]. Thus, there is a pressing need for accessible, non-invasive, and cost-effective biomarkers capable of detecting early-stage AD when intervention may be most beneficial [6].

To address this, attention has increasingly turned to blood-based biomarkers that could support early diagnosis, longitudinal monitoring, and therapeutic stratification [7]. Fluid biomarkers such as plasma phosphorylated tau at threonine 217 (p-tau217), the Aβ42/Aβ40 ratio, neurofilament light chain (NfL), and glial fibrillary acidic protein (GFAP) have been integrated into the AT(N) framework, reflecting amyloid (A), tau (T), and neurodegeneration (N) [8,9]. Among these, p-tau217 is emerging as a leading candidate for identifying amyloid pathology and predicting disease progression [10]. While p-tau217 is highly sensitive for detecting amyloid pathology and preclinical AD [8,10], its elevation may primarily reflect established pathology, potentially limiting its ability to capture the earliest immune-mediated stages of AD [11]. Furthermore, most blood-based biomarkers primarily reflect ongoing neurodegeneration, limiting their utility as early indicators of preclinical AD [12].

In contrast, changes in peripheral immune markers may precede overt neurodegenerative pathology [13]. Among these, syndecan-3 (SDC3)—a transmembrane heparan sulfate proteoglycan (HSPG) expressed in neurons, endothelial cells, and leukocytes—has emerged as a compelling candidate [14,15,16]. SDC3 facilitates cell–matrix interactions, inflammatory signaling, and receptor-mediated endocytosis, and it is known to facilitate the seeding and spreading of misfolded protein aggregates [14,15,17]. Preclinical studies have shown that SDC3 accelerates Aβ aggregation and amyloid plaque seeding in the brain [18]. SDC3 is also overexpressed in neurodegeneration-vulnerable regions of AD brains [19,20]. In APPSWE-Tau transgenic mice, SDC3 was upregulated in both central nervous system and peripheral tissues, including blood-derived monocytes, where its expression correlated with cerebral amyloid burden [16]. These findings suggest that monocyte-expressed SDC3 may serve as an early peripheral biomarker of amyloid pathology and immune activation in AD. Unlike p-tau217, which reflects established amyloid and tau pathology, SDC3 expression may indicate earlier phases of disease development. Moreover, given its expression in circulating immune cells, SDC3 is accessible via minimally invasive blood sampling, making it suitable for routine clinical testing and longitudinal monitoring.

While current biomarker efforts have focused on neuronal and astroglial signatures, relatively little attention has been given to the immune arm of the AD cascade—especially the role of peripheral immune remodeling and leukocyte signaling [21]. Given that neuroinflammation is now considered a key driver of AD pathogenesis, the identification of blood-based immune markers such as SDC3 could provide critical insights into the early and immunological dimensions of the disease [22,23].

Here, we sought to translate our preclinical findings into a clinical setting by evaluating SDC3 expression in peripheral blood mononuclear cells (PBMCs) from patients with clinically diagnosed AD and cognitively unimpaired non-AD controls. To facilitate clinical implementation, we employed a practical ELISA-based method on buffy coat-derived PBMCs rather than purified monocytes. In addition to comparing SDC3 levels between groups, we assessed correlations with plasma p-tau217 and a broad panel of systemic laboratory parameters, including inflammatory, metabolic, and vascular markers. This study evaluates SDC3 expression in PMBCs from patients with clinically diagnosed AD, aiming to establish its potential as a non-invasive biomarker and complementing existing tools in the early detection and stratification of AD.

## 2. Results

### 2.1. Baseline Clinical and Immunological Features of Study Cohorts

Given their role in mediating amyloid and tau interactions via heparan sulfate chains, SDCs have been implicated in the propagation of misfolded proteins in AD [14,15,18]. To evaluate SDC3 expression as a novel biomarker for AD, we first established and compared the clinical and laboratory profiles of two well-defined patient groups: individuals with clinically confirmed AD (AD group, *n* = 22) and a cognitively unimpaired cohort without AD (non-AD group, *n* = 20). A post hoc power analysis confirmed that the total sample size (*n* = 42) provides 81.6% power to detect moderate-to-large effect sizes in group comparisons (Cohen’s *d* = 0.8, α = 0.05), supporting the robustness of the observed group-level differences. Patient classification into AD and non-AD groups was based on documented diagnoses recorded in official medical records. AD diagnoses were established by board-certified neurologists using clinical assessment, standardized cognitive testing, and neuroimaging, in accordance with the NIA-AA 2018 diagnostic guidelines [24].

The AD group was significantly older (81.2 ± 1.5 years) than the non-AD group (59.8 ± 5.0 years). The non-AD group included 40% males, while the AD group had 27.3% males, indicating a slight female predominance in both cohorts; however, this difference in sex distribution was not statistically significant (*p* = 0.3946; Table 1). Both cohorts had comparable body mass indices (BMIs), with means within the normal range (AD: 23.9 ± 0.6 kg/m^2^; non-AD: 24.3 ± 1.5 kg/m^2^).

Beyond demographic distinctions, both groups exhibited substantial comorbidity burdens, reflective of an elderly clinical population (Supplementary Table S1). In the non-AD group, hypertension (50%) and cardiovascular disease (45%) were the most prevalent, followed by diabetes (20%), chronic lung disease (10%), obesity (15%), autoimmune disorders (10%), and gynecological conditions (25%). Neuropsychiatric disease was rare. The AD group displayed a more uniform geriatric comorbidity profile with higher rates of cardiovascular disease (72.7%), hypertension (59.1%), and neuropsychiatric disorders (23%). Insulin-treated diabetes affected 9.1% of patients, while autoimmune, thyroid, and chronic lung diseases occurred each in ~13%. Depression was reported in 18.2%, with isolated cases of schizophrenia and Parkinson’s disease.

Non-AD participants frequently used antihypertensives, proton pump inhibitors, and anxiolytics, while AD patients commonly received cholinesterase inhibitors, NMDA antagonists, anticoagulants, and psychotropics. Neuroprotective supplements and metabolic agents were also common in both groups (Supplementary Table S2).

Mean systolic and diastolic blood pressure were similar across groups (non-AD: 127.67 ± 6.37 vs. AD: 127.14 ± 4.74 mmHg [systolic]; non-AD: 77.0 ± 3.49 vs. AD: 73.32 ± 2.76 mmHg [diastolic]; *p* > 0.4 for both), with greater interindividual variability in the AD cohort. Oxygen saturation (SpO_2_) was maintained (non-AD: 94.84 ± 1.3%; AD: 95.82 ± 0.62%; *p* = 0.48).

Red blood cell indices were comparable between groups, with no statistically significant differences in red blood cell count (non-AD: 4.25 ± 0.14 T/L vs. AD: 4.19 ± 0.14 T/L, *p* = 0.76), hemoglobin concentration (non-AD: 125.0 ± 4.07 g/L vs. AD: 126.68 ± 4.46 g/L, *p* = 0.78), or hematocrit (non-AD: 0.368 ± 0.012 L/L vs. AD: 0.37 ± 0.01 L/L, *p* = 0.76). While not statistically significant, platelet parameters showed a reduction trend in the AD group, including platelet count (non-AD: 234.55 ± 19.82 G/L vs. AD: 206.18 ± 22.16 G/L, *p* = 0.35) and mean platelet volume (non-AD: 11.04 ± 0.25 fL vs. AD: 10.9 ± 0.22 fL, *p* = 0.72). The total white blood cell (WBC) count did not differ significantly (non-AD: 11.62 ± 1.65 G/L vs. AD: 8.02 ± 0.93 G/L, *p* = 0.059), yet key leukocyte subsets revealed significant shifts suggestive of systemic immune remodeling in AD. The monocyte percentage was significantly elevated in the AD group (non-AD: 6.9 ± 0.4% vs. AD: 10.3 ± 0.8%, *p* = 0.002), while eosinophil (non-AD: 0.79 ± 0.22% vs. AD: 0.24 ± 0.14%, *p* = 0.046) and basophil percentages (non-AD: 0.363 ± 0.041% vs. AD: 0.23 ± 0.035%, *p* = 0.020) were significantly reduced. These findings are consistent with low-grade chronic inflammation, innate immune activation, and immunosenescence typically observed in AD (Table 2) [25,26].

Circulating inflammatory markers differed notably between groups. C-reactive protein (CRP) was significantly higher in non-AD (114.86 ± 27.50 mg/L) than in AD patients (43.69 ± 9.68 mg/L, *p* = 0.015) despite AD’s link to chronic neuroinflammation. IL-6 levels also trended higher in the non-AD group (non-AD: 173.9 ± 63.66 pg/mL vs. AD: 62.47 ± 11.9 pg/mL, *p* = 0.12), though variability limited significance. This likely reflects transient or subclinical inflammatory conditions in the non-AD group unrelated to AD, whereas the AD cohort exhibits a more chronic, compartmentalized inflammatory profile.

Unlike alkaline phosphatase (ALP) and gamma-glutamyltransferase (GGT), which did not differ significantly between groups (*p* = 0.33 and *p* = 0.62, respectively), other liver enzymes were consistently higher in the non-AD group: GOT (non-AD: 42.63 ± 6.34 vs. AD: 27.05 ± 2.01 U/L; *p* = 0.017), GPT (30.42 ± 4.07 vs. 17.68 ± 2.14 U/L; *p* = 0.0064), and LDH (non-AD: 318.95 ± 30.0 vs. AD: 224.05 ± 13.24 U/L; *p* = 0.0043). This pattern may reflect greater metabolic activity, subclinical hepatic strain, or higher muscle mass in the non-AD cohort. In contrast, the lower enzyme levels observed in the AD group could indicate sarcopenia, malnutrition, or reduced systemic turnover.

Cardiac biomarkers displayed substantial interindividual variability in both cohorts. Notably, proBNP levels were significantly elevated in the non-AD group (non-AD: 6727.23 ± 2478.56 pg/mL vs. AD: 1495.14 ± 480.53 pg/mL, *p* = 0.045), suggesting ongoing myocardial stress. Procalcitonin (PCT) levels were also markedly higher in the non-AD patients (non-AD: 4.20 ± 1.98 ng/mL vs. 0.21 ± 0.07 ng/mL in AD, *p* = 0.046), likely reflecting acute-phase conditions not present in the AD cohort.

Collectively, these findings indicate that while both cohorts displayed clinical and laboratory evidence of chronic disease and systemic stress, the AD group is distinguished by a distinct immunological profile characterized by elevated circulating monocytes and reduced eosinophil and basophil percentages. This leukocyte signature aligns with known patterns of innate immune activation and neuroinflammation in AD. Importantly, these parameters provide a robust biological context and critical baseline for interpreting the diagnostic relevance of PBMC-expressed SDC3 as a novel, disease-specific biomarker for AD.

### 2.2. Elevated p-tau217 in Non-AD Patients Linked to Systemic Inflammation

**Figure 1:**
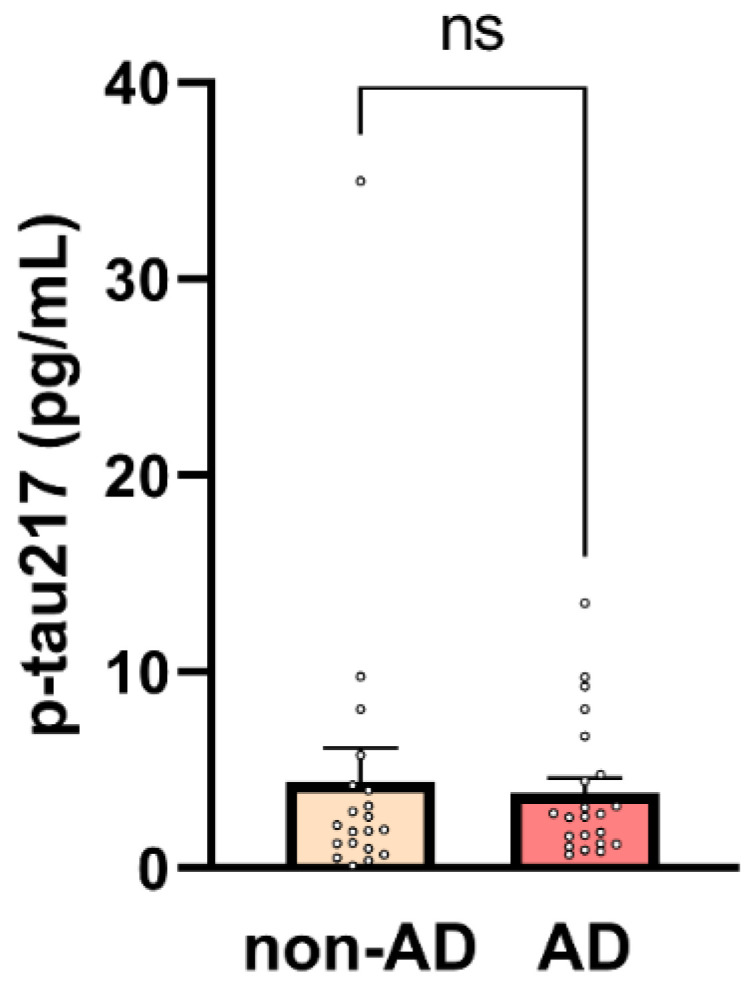
Plasma p-tau217 concentrations in non-AD and AD participants. Mean ± SEM plasma p-tau217 levels are shown for non-AD (*n =* 20) and AD (*n* = 22) participants. Each dot represents an individual participant. No significant difference was observed between groups (unpaired two-tailed *t*-test, *p* = 0.75, ns = non-significant).

Phosphorylated tau (p-tau) has demonstrated strong potential as a biomarker of amyloid pathology, as indicated by its association with CSF and PET amyloid positivity [10]. Various p-tau isoforms, defined by distinct phosphorylation sites, have been recognized for their ability to differentiate amyloid-positive individuals. Among these, p-tau217 has emerged as a particularly robust indicator of amyloid pathology in AD, demonstrating high diagnostic accuracy and potential for early detection [10]. However, in our study, p-tau217 levels were unexpectedly elevated in the non-AD cohort, resulting in no statistically significant difference in p-tau217 concentrations between the AD and non-AD groups. As shown in Figure 1, the mean p-tau217 value was higher in the non-AD group, although this difference did not reach statistical significance (non-AD: 4.42 ± 1.71 pg/mL vs. 3.84 ± 0.74 pg/mL in AD, *p* = 0.75).

**Figure 2:**
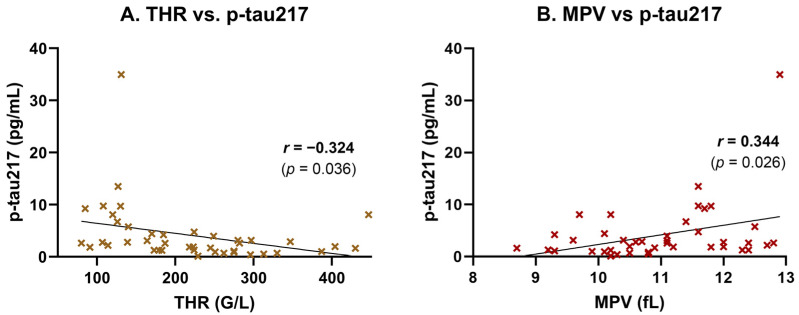
Correlation of plasma p-tau217 with platelet parameters. Plasma p-tau217 concentrations were correlated with platelet indices in participants (*n* = 42). (**A**) p-tau217 showed a moderate negative correlation with THR (*r* = −0.32, *p* = 0.036), suggesting that higher p-tau217 concentrations are associated with lower platelet counts. (**B**) p-tau217 exhibited a moderate positive correlation with MPV (*r =* 0.34, *p =* 0.02570), suggesting potential platelet activation in participants with higher p-tau217 concentrations. Scatter plots show individual data points, linear regression lines, and Pearson correlation coefficients (*r*) with *p*-values.

To explore the unexpectedly high p-tau217 levels observed among non-AD subjects and assess potential systemic influences, we performed Pearson correlation and linear regression analyses between plasma p-tau217 concentrations and routine laboratory parameters. A moderate, statistically significant negative correlation was found between p-tau217 and platelet count (THR) (*r* = −0.324, *p* = 0.036), indicating that higher p-tau217 levels tend to be associated with lower thrombocyte counts (Figure 2A). This association was supported by simple linear regression (Table 3). Conversely, the mean platelet volume (MPV) showed a moderate, statistically significant positive correlation with p-tau217 *(r* = 0.344, *p* = 0.026), suggesting that individuals with elevated p-tau217 tend to exhibit a larger average platelet size—a potential marker of platelet activation or turnover (Figure 2B). This relationship was also confirmed by linear regression (Table 3).

**Figure 3:**
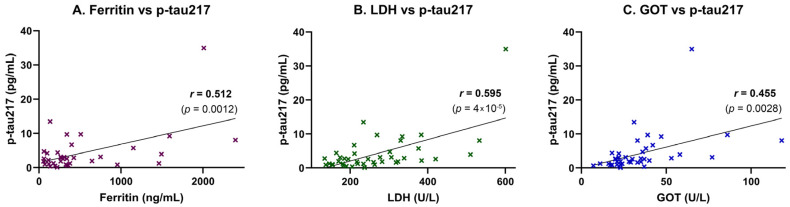
Correlation of plasma p-tau217 with inflammatory and hepatic stress biomarkers. Plasma p-tau217 concentrations were correlated with markers of systemic inflammation, iron metabolism, and hepatic stress in study participants (*n* = 42). Scatter plots show individual data points, linear regression lines, and Pearson correlation coefficients (*r*) with corresponding *p*-values. (**A**) Ferritin correlated moderately with p-tau217 (*r* = 0.512, *p* = 0.0012), suggesting a link to systemic inflammation or iron dysregulation. (**B**) LDH showed a strong positive correlation (*r* = 0.595, *p* < 0.00004), consistent with tissue damage and systemic inflammation. (**C**) GOT exhibited a moderate positive correlation (*r* = 0.455, *p* = 0.0028), indicating potential hepatic involvement.

Inflammatory and tissue injury markers were also associated with plasma p-tau217 levels. A moderate, statistically significant positive correlation was observed between p-tau217 and ferritin (*r* = 0.512, *p* = 0.0012), suggesting a link to systemic inflammation or iron dysregulation (Figure 3A). This association was supported by simple linear regression analysis, which confirmed ferritin as a significant predictor of p-tau217 concentrations (*p* = 0.0012; Table 4). A strong and highly significant positive correlation was also found between p-tau217 and lactate dehydrogenase (LDH), a marker of cellular damage and systemic inflammation (*r* = 0.595, *p* = 0.00004; Figure 3B). This relationship was likewise confirmed in the regression model, with LDH significantly predicting p-tau217 levels (*p* < 0.0001; Table 4). Additionally, p-tau217 levels were moderately and significantly correlated with aspartate aminotransferase (GOT/AST) (*r* = 0.455, *p* = 0.0028; Figure 3C), and this association also remained significant in the linear regression analysis (*p* = 0.0028; Table 4).

Together, these findings suggest that plasma p-tau217 levels may partly reflect systemic hepatic or inflammatory processes—particularly in comorbid, non-AD individuals—underscoring the need for contextual interpretation in elderly populations.

### 2.3. SDC3 Expression in Peripheral Blood Leukocytes Is Elevated in AD Patients

**Figure 4:**
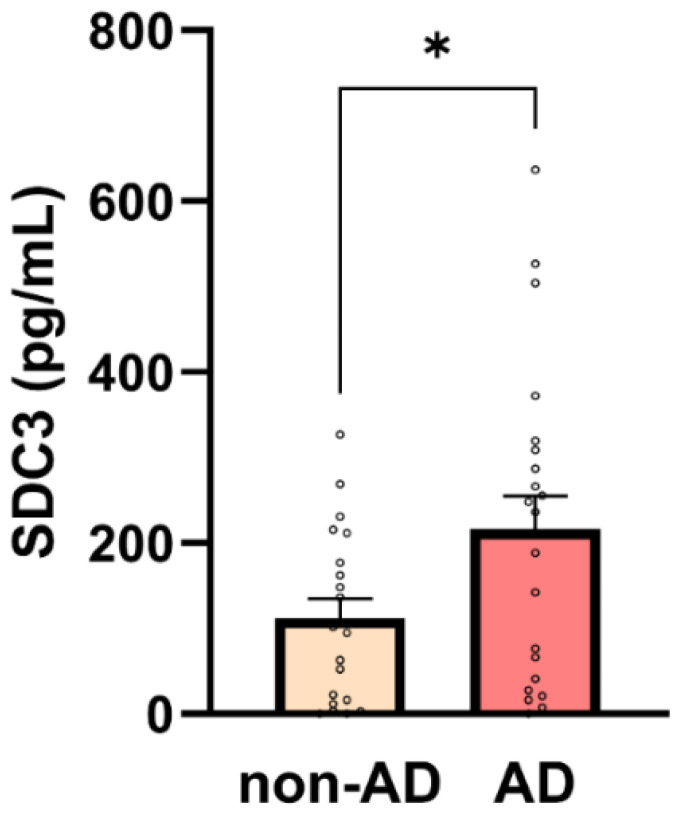
SDC3 concentrations in PBMCs are higher in AD participants. Bar plots show mean + SEM SDC3 concentrations in peripheral blood mononuclear cells (PBMCs; 1 × 10^5^ cells/sample) for non-AD (*n* = 20) and AD (*n* = 22) participants with individual data points. SDC3 concentrations were significantly higher in AD vs. non-AD participants (unpaired two-tailed *t*-test, * *p* = 0.03).

Simultaneously with p-tau217, we also analyzed the expression of SDC3 in blood-derived PBMCs. Unlike in our previous preclinical studies, we did not isolate monocytes specifically; instead, we obtained the PBMC fraction through density gradient centrifugation (e.g., using density gradient medium) and isolating the PBMC-containing buffy coat. This methodological simplification was intended to assess whether SDC3 measurement in a PBMC sample—prepared using a basic centrifugation step feasible in resource-limited hospital settings—could effectively distinguish AD patients from non-AD controls. Thus, PBMCs were isolated, and 1 × 10^5^ cells per sample were used to measure SDC3 expression with a custom SDC3 ELISA. As shown in Figure 4, SDC3 levels in PBMCs from AD patients were significantly higher than in non-AD individuals (216.51 ± 38.75 pg/mL vs. 112.41 ± 22.75 pg/mL; *p* = 0.03).

A moderate, statistically significant correlation was observed between SDC3 expression and AD status (*r* = 0.309, *p* = 0.0465). To evaluate its predictive value, we performed univariable logistic regression using SDC3 levels as the sole predictor of AD diagnosis. The model was statistically significant (*p* = 0.0409), with SDC3 emerging as a significant predictor (β = 0.00517 ± 0.00253; *p* = 0.0409), corresponding to an odds ratio (OR) of 1.005 (95% CI: 1.001–1.011). This implies that each unit increase in SDC3 expression was associated with a 0.5% increase in the odds of having AD. The model’s performance was modest, with an area under the ROC curve (AUC) of 0.682 (95% CI: 0.519–0.844; *p* = 0.0439), indicating fair discriminative ability (Table 5).

### 2.4. Correlation of PBMC-Expressed SDC3 with Age and Gender

Given the age difference between the AD and control participants, we analyzed whether PBMC-expressed SDC3 levels were associated with age. However, no significant correlation was observed (*r* = 0.2090, *p* = 0.1841), suggesting that the elevated SDC3 levels in AD patients are not simply attributable to chronological aging. This was further supported by a simple linear regression analysis (slope = 1.686, 95% CI: −0.835 to 4.206, *p* = 0.1841), which did not identify age as a significant predictor of SDC3 expression (R^2^ = 0.044). These findings indicate that age alone does not account for the observed differences in leukocyte SDC3 levels.

**Figure 5:**
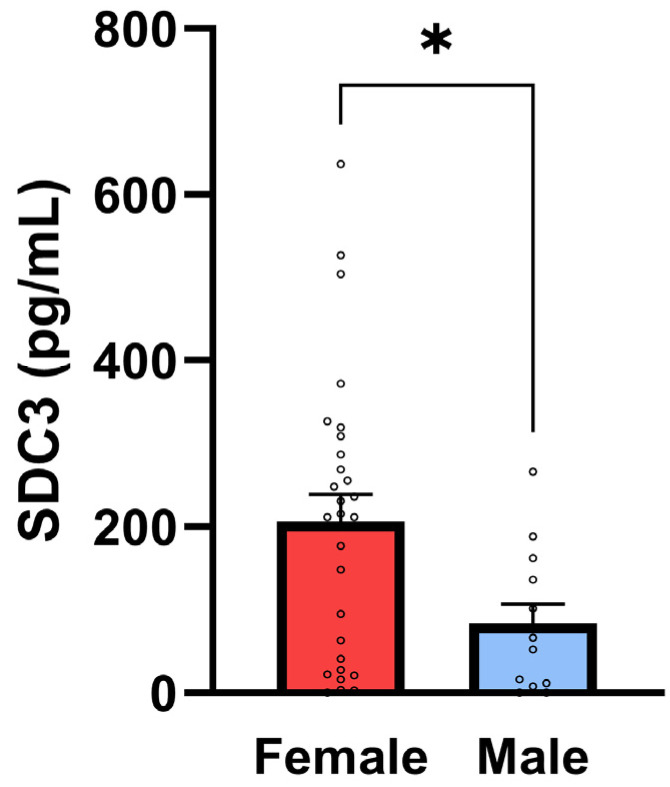
SDC3 concentrations in PBMCs are higher in female participants. Bar plots show mean ± SEM SDC3 concentrations in PBMCs (1 × 10^5^ cells/sample) for female (*n* = 28) and male (*n* = 14) participants with individual data points. SDC3 concentrations were significantly higher in female vs. male participants (unpaired two-tailed *t*-test, * *p* = 0.02).

A moderate and statistically significant positive correlation was identified between sex and SDC3 expression (*r* = 0.362, *p* = 0.0184), indicating higher SDC3 levels in females compared to males in the overall cohort (with sex coded as a binary variable; Figure 5). This sex-based difference may reflect hormonal or epigenetic modulation of SDC3 expression and warrants further investigation.

However, when stratified by diagnostic status, the sex-related differences in SDC3 expression were no longer statistically significant within either the non-AD or AD subgroups (Table 6). These results support the diagnostic potential of PBMC-expressed SDC3 in AD and underscore the importance of further investigating sex-specific patterns and their biological underpinnings in larger, balanced cohorts.

### 2.5. Correlation of PBMC-Expressed SDC3 with Systemic Laboratory Markers

**Figure 6:**
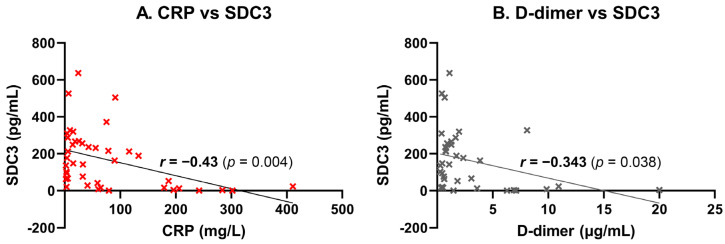
Correlation of leukocyte-expressed SDC3 with systemic laboratory markers. SDC3 expression in peripheral blood leukocytes was correlated with systemic markers in participants (*n* = 42). Scatter plots show individual data points, linear regression lines, and Pearson correlation coefficients (*r*) with *p*-values. (**A**) CRP concentrations showed moderate negative correlation (*r* = −0.43, *p* = 0.004), suggesting reduced SDC3 expression with elevated inflammation. (**B**) D-dimer concentrations correlated weakly and negatively (*r* = −0.34, *p* = 0.038), potentially indicating dissociation from vascular injury.

PBMC-expressed SDC3 exhibited a moderate, statistically significant negative correlation with systemic CRP levels (*r* = −0.43, *p* = 0.004), suggesting that higher SDC3 expression may be associated with lower levels of systemic inflammation (Figure 6A). This inverse relationship may indicate that SDC3 expression marks a distinct, potentially regulatory immune phenotype that is less responsive to acute-phase inflammatory stimuli. Similarly, a significant negative correlation was observed between SDC3 and D-dimer levels (*r* = −0.343, *p* = 0.038), potentially implying that increased SDC3 expression reflects an immune activation state that is independent of systemic coagulopathy or vascular injury (Figure 6B). Collectively, these results support the hypothesis that leukocyte-expressed SDC3 represents a non-canonical immune state associated with AD, distinct from classical markers of inflammation, coagulation, or vascular pathology.

### 2.6. SDC3 Expression in the Context of Antihypertensive Therapy

When analyzing its correlation with comorbidities, PBMC-expressed SDC3 showed a moderate, statistically significant inverse correlation with diagnosed hypertension status (*r* = −0.335, *p* = 0.0299), indicating that hypertensive individuals tended to exhibit lower SDC3 levels. At first glance, this appears paradoxical, given the well-established vascular burden and inflammatory stress associated with hypertension. However, a comparison of measured blood pressure values between hypertensive and normotensive individuals revealed no significant differences in either systolic or diastolic blood pressure (Table 7).

This finding suggests that hypertension classification was not solely based on contemporaneous blood pressure readings but likely reflected prior medical diagnosis and ongoing antihypertensive treatment. The pharmacological profile of the hypertensive group supports this interpretation: most patients were receiving medications with previously reported neuroprotective effects in AD [27,28,29]. These included ACE inhibitors (e.g., perindopril, and enalapril), angiotensin receptor blockers (e.g., telmisartan), calcium channel blockers (e.g., amlodipine and felodipine), and beta-blockers (e.g., carvedilol and nebivolol), as well as anticoagulants such as apixaban and rivaroxaban [30,31,32,33,34]. Several of these agents have been associated with reduced neuroinflammation, improved blood–brain barrier function, and attenuation of amyloid or tau pathology in both preclinical and clinical studies [29,35,36,37,38]. Accordingly, the lower SDC3 expression observed in hypertensive individuals may reflect the immunomodulatory or neuroprotective effects of chronic antihypertensive therapy rather than the pathological consequences of hypertension itself.

### 2.7. Diagnostic Performance of SDC3 Expression in PBMCs

**Figure 7:**
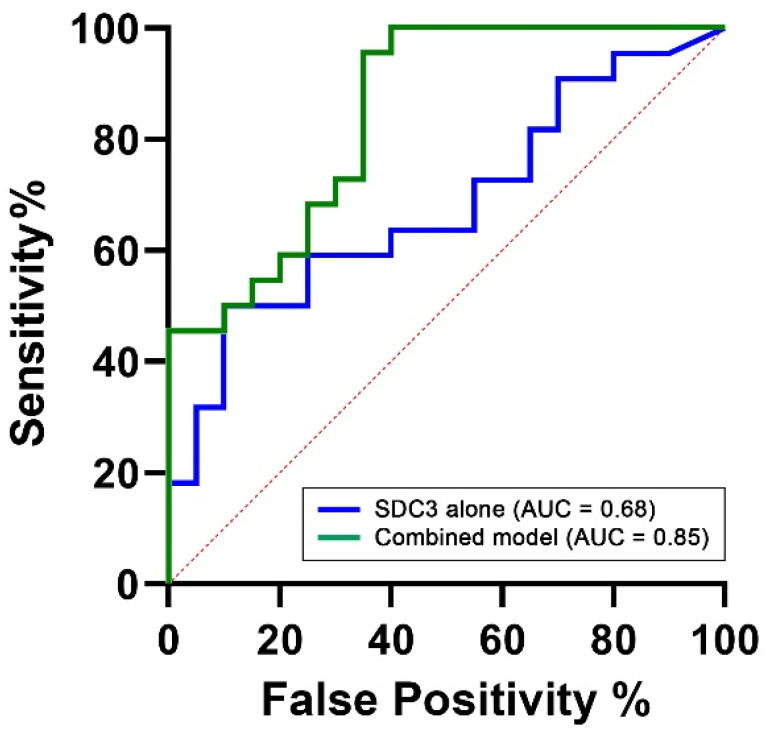
ROC curves comparing the diagnostic performance of PBMC-expressed SDC3 alone versus a multivariable model. The ROC curves illustrate the classification performance of SDC3 alone (blue) and the multivariable logistic regression model (green), which includes SDC3, plasma p-tau217, and age. The SDC3-only model yielded an area under the curve (AUC) of 0.68, indicating moderate diagnostic performance. In contrast, the multivariable model achieved an AUC of 0.85, reflecting excellent discrimination between AD and non-AD participants. The x-axis represents the false positive rate (1 − specificity), and the y-axis represents the true positive rate (sensitivity).

To assess the diagnostic performance of PBMC-expressed SDC3, we performed a receiver operating characteristic (ROC) analysis using SDC3 levels as a standalone predictor of AD status. This analysis yielded an area under the curve (AUC) of 0.68 (95% CI: 0.52–0.84, *p* = 0.044; Table 5), indicating moderate discriminatory power between AD and non-AD participants (Figure 7). This is encouraging, considering the limited cohort size and the intrinsic variability associated with peripheral immune markers.

To determine whether diagnostic performance could be improved through the inclusion of complementary biomarkers, we constructed a multivariable logistic regression model. Predictors were selected based on biological plausibility and prior evidence. PBMC-expressed SDC3 was retained as the central biomarker of interest. Age was included due to its well-established and strong association with AD risk across all diagnostic models (AUC = 0.78, 95% CI: 0.63–0.93, *p* = 0.002). Plasma p-tau217 was included as a key neurodegeneration-related biomarker, reflecting tau pathology and representing one of the most clinically validated blood-based indicators of AD. However, in our cohort, p-tau217 alone demonstrated limited discriminatory capacity (AUC = 0.55, 95% CI: 0.38–0.73, *p* = 0.5624). The final multivariable model, integrating SDC3, p-tau217, and age, achieved an AUC of 0.85 (95% CI: 0.73–0.96, *p* = 0.0001), with a Nagelkerke R^2^ value of 0.54, indicating excellent overall discriminative ability. This value suggests that the model explains approximately 54% of the variance in AD status, reflecting a strong overall model fit. Such a level of explanatory power is particularly noteworthy in the context of a complex, multifactorial disease like AD, where the integration of immunological, neurodegenerative, and demographic markers can meaningfully enhance diagnostic accuracy.

## 3. Discussion

This study identifies SDC3 expression in PBMCs as a novel and potentially disease-relevant biomarker for AD. Our findings show that SDC3 levels are significantly elevated in AD patients compared to cognitively unimpaired, non-AD individuals and demonstrate diagnostic relevance as a peripheral biomarker for AD. Importantly, SDC3 expression appears to reflect an AD-associated immunological signature that differs from classical systemic inflammatory or vascular responses.

While most established AD biomarkers focus on central amyloid and tau pathology, peripheral immune remodeling is increasingly recognized as a critical contributor to neurodegenerative progression [1,13]. In line with this, we observed distinct alterations in circulating immune cell profiles in AD patients, including increased monocyte proportions and reduced eosinophil and basophil percentages—consistent with innate immune activation and immunosenescence commonly reported in AD [25,26,39].

An unexpected but noteworthy observation was the lack of significant elevation in plasma p-tau217 levels in the AD group. In fact, some non-AD participants exhibited high p-tau217 values, and correlation analyses revealed associations between p-tau217 and systemic inflammation (e.g., CRP and ferritin), hepatocellular stress (e.g., LDH, GOT, and GPT), and altered platelet dynamics. These findings imply that in elderly, comorbid populations, p-tau217 levels may be influenced not only by CNS tau pathology but also by systemic physiological stressors—potentially limiting its diagnostic specificity under certain clinical conditions. This interpretation is supported by recent large-scale evidence from Mielke et al., who demonstrated that multiple comorbidities, including chronic kidney disease, hypertension, myocardial infarction, and stroke, are independently associated with elevated plasma p-tau181 and p-tau217, even in amyloid-negative individuals [40]. These comorbidities likely affect plasma tau levels via impaired clearance or systemic stress, highlighting the risk of false positives in multimorbid populations. Additionally, Abu-Rumeileh et al. reported elevated plasma p-tau217 in patients with amyotrophic lateral sclerosis (ALS), potentially originating from denervated muscle fibers [41]. Together, these studies suggest that tau metabolism and plasma levels can be modulated by extra-cerebral factors, particularly in aging individuals with multiple comorbidities. This challenges the assumption that plasma p-tau181 and p-tau217 are exclusively CNS-derived and raises important concerns regarding their accuracy as standalone screening tools for AD pathology in complex clinical settings.

It is important to note that the absolute p-tau217 values observed in our study were measured using a commercially available sandwich ELISA with a validated detection range of 0.5–32 pg/mL. This contrasts with lower values typically reported in studies employing ultrasensitive platforms such as Simoa or mass spectrometry, which yield sub-picogram concentrations due to differences in detection limits, standardization, and calibration curves [42,43]. These methodological differences underscore the need for international assay harmonization to enable standardized reference ranges and improve cross-study interpretability.

In contrast, PBMC-expressed SDC3 showed a consistent inverse relationship with inflammatory and vascular markers, including CRP and D-dimer. These findings strongly suggest that elevated SDC3 expression does not reflect general inflammatory activity or acute-phase responses. Instead, SDC3 appears to represent a distinct, non-inflammatory immune signature associated with chronic neurodegenerative pathology. This decoupling from systemic inflammation reinforces SDC3’s potential diagnostic resilience in diverse clinical contexts.

The immunomodulatory function of SDC3 may underlie this profile [44]. As a transmembrane heparan sulfate proteoglycan involved in leukocyte trafficking, cytokine signaling, and barrier regulation, SDC3 contributes to fine-tuning immune homeostasis and neuroimmune communication [45,46,47,48]. Its elevation in AD may reflect a disease-specific shift in peripheral immune tone rather than nonspecific immune activation, supporting its use as a more refined and stable marker of neurodegenerative immune remodeling.

The inverse association between SDC3 and hypertension status further reinforces this interpretation. Although hypertension is a known AD risk factor, SDC3 levels were significantly lower in hypertensive individuals despite no difference in measured blood pressure values. Many hypertensive participants were on long-term antihypertensive medications with proposed neuroprotective effects, such as ACE inhibitors, angiotensin receptor blockers, beta-blockers, and calcium channel blockers [27,28,29,30,31,32,33,34]. This pharmacological context suggests that lower SDC3 expression may reflect treatment-related modulation of immune tone rather than hypertension-induced vascular stress, highlighting the importance of clinical context when interpreting immune biomarkers.

Sex-specific differences in SDC3 expression also emerged: females showed higher overall SDC3 levels than males. Although this difference was not statistically significant within diagnostic subgroups and age showed no correlation with SDC3 expression, these findings suggest that the observed elevation in PBMC-expressed SDC3 among AD patients is not attributable to demographic factors but more likely reflects disease-specific immune remodeling. Given the known influence of sex and age on AD progression and immune regulation, these trends warrant further investigation. Stratification by sex and age in future cohorts may help elucidate their potential interaction with SDC3 expression. [49,50,51]. Moreover, our findings showing an inverse relationship between SDC3 expression and inflammatory markers, including CRP, argue against the possibility that demographic factors associated with inflammation—such as age or sex—are the primary drivers of SDC3 elevation. If SDC3 was predominantly influenced by general inflammation or age-related immune activation, higher levels would be expected in the younger, more inflamed non-AD group. Instead, we observed the opposite: SDC3 was significantly elevated in older AD patients with lower systemic inflammatory burden. These results suggest that SDC3 reflects a disease-specific immune signature rather than nonspecific inflammatory variation.

Importantly, while SDC3 alone demonstrated moderate diagnostic performance (AUC = 0.68), its integration into a multivariable model alongside plasma p-tau217 and age significantly improved classification accuracy (AUC = 0.85, 95% CI: 0.73–0.96, *p* = 0.0001). This synergistic enhancement suggests that SDC3 provides complementary, non-redundant information when combined with established neurodegenerative and demographic markers. These findings indicate that PBMC-expressed SDC3 offers meaningful diagnostic value on its own and substantially enhances classification performance in combination with other biomarkers. This supports its potential as a cornerstone of peripheral blood–based diagnostic panels for AD and underscores the feasibility of developing accessible, multicomponent assays for early and accurate detection.

From a practical perspective, SDC3 measurement requires only basic blood collection and ELISA-based quantification, making it feasible for use in standard hospital laboratories or low-resource settings. Unlike CSF- or PET-based diagnostics, which are invasive or expensive, a blood-based SDC3 assay could support broader screening or preclinical-stage risk stratification efforts.

It is also essential to emphasize that the non-AD group in this study was not a healthy control cohort. Rather, these patients were representative of real-world clinical populations—multimorbid, aged, and burdened with diverse chronic conditions but free of clinically diagnosed AD. This makes the distinction in SDC3 expression even more meaningful as it highlights the biomarker’s ability to differentiate AD from other pathological backgrounds that share overlapping systemic features. The observed age difference between the groups (AD: 81.2 ± 1.5 years vs. non-AD: 59.8 ± 5.0 years) reflects the clinical reality of AD. Due to logistical constraints, cognitively unimpaired elderly individuals without AD were not readily available for this study, making age matching infeasible in this real-world clinical sampling context. While the non-AD cohort included multimorbid non-AD patients, the design reflects real-world diagnostic complexity. Future studies may incorporate stratification or exclusion frameworks to further validate SDC3’s diagnostic performance.

This study has several limitations, most notably the relatively small sample size. Nonetheless, we applied rigorous and appropriate statistical methods to mitigate the constraints typically associated with limited sample sizes. A post hoc power analysis confirmed that the sample size (*n* = 42) provides 81.6% power to detect moderate to large effect sizes in group comparisons (Cohen’s *d* = 0.8, α = 0.05). Nonetheless, the statistical power to detect smaller or more subtle effects—particularly in multivariable models—may be limited. Moreover, the relatively small cohort constrains external generalizability and highlights the need for validation in larger independent populations. The cross-sectional design precludes the evaluation of longitudinal biomarker trajectories, and our ELISA approach does not offer cell-type specificity within the PBMC fraction. Although monocytes are likely the primary source of SDC3, future studies employing flow cytometry or single-cell transcriptomics are needed to confirm this and to map functional SDC3 expression across immune cell subsets.

In conclusion, our findings support PBMC-expressed SDC3 as a promising, accessible, and biologically relevant blood-based biomarker for AD. It appears to capture a dimension of immune remodeling that is distinct from traditional inflammatory signals and complements existing markers of neurodegeneration. By reflecting immune-related or potentially early pathological processes, SDC3 may serve as a valuable addition to multicomponent biomarker panels. Further validation in longitudinal and multi-center cohorts is needed, along with mechanistic studies to explore SDC3’s potential role as both a biomarker and therapeutic target in the AD immune landscape.

## 4. Materials and Methods

### 4.1. Study Population and Data Collection

This study was approved by the Regional and Institutional Review Board of the University of Szeged (approval number: 99/2022-SZTE RKEB; approval date: 29 August 2022). The protocol was reviewed from both ethical and scientific perspectives in accordance with Decree 23/2002 of the Hungarian Ministry of Health and Government Decree 235/2009 (X.20.) and was conducted in compliance with the Declaration of Helsinki. All participants provided written informed consent prior to enrollment. Data were collected and processed in anonymized form to ensure participant confidentiality.

The sample size (*n* = 42; 22 AD, 20 non-AD) was determined based on feasibility for this exploratory study. A post hoc power analysis using G*Power (v3.1.9.7, Heinrich Heine University Düsseldorf, Germany) indicated that this cohort provided 81.6% power to detect a moderate-to-large effect size (Cohen’s *d* = 0.8) in SDC3 level differences at a significance level of α = 0.05 [52,53].

Clinical, neuropsychological, and comorbidity information was extracted from hospital records. Demographic and diagnostic data were collected with a specific focus on AD. AD diagnoses were established based on internationally recognized criteria—such as those of the National Institute on Aging—Alzheimer’s Association (NIA-AA) and the International Working Group (IWG), as applied in the Hungarian clinical context [24,54,55].

In addition to AD diagnosis, key demographic variables such as age, sex, and BMI were recorded. Comorbidity data were also systematically collected, including the presence of hypertension, cardiovascular diseases, chronic lung diseases, psychiatric or mental health disorders, and diabetes mellitus, among others.

Peripheral blood samples were collected and subjected to standard laboratory analyses, including complete blood counts, inflammatory markers, hepatic and cardiac enzymes, and selected AD-related biomarkers.

To ensure data integrity, all clinical and laboratory entries were independently reviewed and cross-validated by two researchers. This dual-check process minimized potential biases and supported the robustness of subsequent statistical analyses.

### 4.2. Plasma Preparation and Storage

For plasma collection, 3 mL of venous blood was drawn into sterile EDTA-coated tubes (e.g., VACUETTE^®^ purple-top tubes, cat. no. 454021, Greiner Bio-One, Kremsmünster, Austria) and processed immediately to prevent coagulation. Samples were centrifuged at 2000× *g* for 10 min at 4 °C to separate plasma from cellular components. The resulting supernatant (plasma) was carefully aspirated with a calibrated pipette and transferred into sterile microcentrifuge (Eppendorf, Hamburg, Germany) tubes, minimizing contamination and hemolysis. For storage, 0.5 mL aliquots were prepared and stored at −20 °C or below until analysis.

### 4.3. Isolation of PBMCs from Whole Blood

For the isolation of PBMCs, 6.0 mL of whole blood was collected and placed into EDTA tubes. To a 15 mL conical centrifuge tube, 6.0 mL of HISTOPAQUE^®^-1077 (cat. no. H8889, Sigma-Aldrich, St. Louis, MO, USA) was added and allowed to reach room temperature. The 6.0 mL sample of whole EDTA blood was then carefully layered onto HISTOPAQUE^®^-1077. The sample was centrifuged at 4000× *g* for exactly 40 min at room temperature. It is important to avoid centrifugation at lower temperatures, such as 4 °C, as this can cause cell clumping and result in poor recovery.

After centrifugation, the upper plasma layer was carefully aspirated using a Pasteur pipette, ensuring the aspirated liquid was within 0.5 cm of the opaque interface (the “Buffy coat”) containing the mononuclear cells. The plasma was collected into Eppendorf tubes and frozen at −20 °C for further analysis. The buffy coat was then transferred into a clean conical centrifuge tube using a Pasteur pipette. To this tube, 1 mL of sterile phosphate-buffered saline (PBS) was added and mixed gently.

The mixture was centrifuged at 300× *g* for 10 min. After centrifugation, the supernatant was aspirated and discarded. The cells were fixed by adding 500 µL of 4% paraformaldehyde (PFA, cat. no. J61899.AK, Thermo Fisher Scientific, Waltham, MA, USA) to the tube and incubating at room temperature for 10 min. Following fixation, 1 mL of sterile PBS was added to the tube and gently mixed. The sample was centrifuged again at 300× *g* for 10 min, the supernatant was discarded, and the cells were resuspended in 1 mL of PBS and stored at 4 °C.

### 4.4. Human Plasma p-tau217 ELISA Measurements

To quantify human plasma phospho-Tau (Thr217) levels, we employed a sandwich enzyme-linked immunosorbent assay (ELISA) using the Human Phospho-Tau (Thr217) ELISA Kit from MyBioSource (cat. no. MBS1608795, San Diego, CA, USA). The 96-well microplate provided in the kit was pre-coated with an antibody specific to human p-tau217.

Plasma samples were diluted to a ratio of 1:10 using the standard assay diluent provided with the kit, in accordance with the manufacturer’s instructions, to ensure accurate quantification and prevent signal saturation. During the assay, 50 µL of standard solution was added to the designated standard wells. In sample wells, 40 µL of the diluted plasma sample and 10 µL of biotinylated anti-p-tau217 antibody were added, followed by 50 µL of streptavidin-HRP conjugate to all wells except the blank. The plate was incubated at 37 °C for 60 min. After incubation, the wells were washed five times with wash buffer, and 50 µL each of substrate solutions A and B were added to all wells. The plate was then incubated in the dark for 10 min. The reaction was terminated with stop solution, inducing a color change from blue to yellow, and optical density (OD) was measured at 450 nm within 10 min using a BioTek Cytation 3 multimode microplate reader (BioTek Instruments, Winooski, VT, USA).

All standards and samples were handled uniformly and analyzed in duplicate. The reported results represent the average of these parallel measurements. No signal oversat-uration or compression was detected, and all values were within the assay’s validated dynamic range. Variability between replicates consistently remained below 20%, support-ing the assay’s reliability (Supplementary Table S3).

### 4.5. ELISA Measurements for SDC3 Quantification in PBMC Samples

SDC3 levels in PBMCs were quantified using a sandwich enzyme-linked immunosorbent assay (ELISA) with the human SDC3 ELISA Kit (cat. no. ABIN4884706, Antibodies-online Inc., Pottstown, PA, USA) according to the manufacturer’s instructions. Equal cell numbers (1 × 10^5^ cells/well) were used across all samples to ensure consistency and prevent signal saturation. PBMCs were isolated from whole blood via density gradient centrifugation using HISTOPAQUE^®^-1077 (Sigma-Aldrich, cat. no. H8889), followed by two washes with phosphate-buffered saline (PBS; pH 7.4).

For the ELISA, 1 × 10^5^ PBMCs and 100 µL of standard solution were added per well in a pre-coated 96-well microplate and incubated for 2.5 h at room temperature. After washing the wells three times with wash buffer, 100 µL of biotin-conjugated anti-SDC3 detection antibody was added, followed by a 1 h incubation period at room temperature. Wells were then washed again, and 100 µL of streptavidin–horseradish peroxidase (HRP) conjugate was added and incubated for 45 min.

After additional washing steps, 100 µL of TMB substrate solution was added and the enzymatic reaction was developed in the dark for 30 min at room temperature. The reaction was stopped by adding 50 µL of stop solution, and absorbance was measured immediately at 450 nm using a BioTek Cytation 3 multimode microplate reader.

All samples and standards were processed identically and measured in duplicate. Reported values reflect the means of parallel measurements. No oversaturation or signal compression was observed, and all values fell within the validated dynamic range of the assay. Inter-replicate deviations remained consistently below 20%, confirming assay reliability. Replicate data are summarized in Supplementary Table S3.

### 4.6. Statistical Analyses

Data are presented as mean ± standard error of the mean (SEM). Group comparisons were performed using unpaired two-tailed *t*-tests, with *p*-values < 0.05 considered statistically significant. All statistical analyses and visualizations were conducted using IBM^®^ SPSS^®^ Statistics version 26 and GraphPad Prism version 9.

Pearson correlation analyses were used to assess associations between plasma p-tau217 and systemic inflammatory, hepatic, and hematologic markers.

To evaluate the diagnostic potential of PBMC-expressed SDC3, ROC analyses were conducted. Binary logistic regression was used to model AD status (1 = AD; 0 = non-AD) as the dependent variable, with SDC3, plasma p-tau217, and age included as predictors. The diagnostic performance of univariable and multivariable models was compared based on AUC values.

A post hoc power analysis, conducted using G*Power (v3.1.9.7), confirmed that the sample size provided 81.6% power to detect a moderate to large effect size (Cohen’s *d* = 0.8) in group comparisons at a significance level of α = 0.05.

## Figures and Tables

**Table 1:** Demographic comparison of the non-AD vs. AD groups.

Parameter	Non-AD (Mean ± SEM)	AD (Mean ± SEM)	*p*-Value
Age (years)	59.8 ± 5.0	81.2 ± 1.5	<0.0001
Sex (% male)	40%	27.3%	0.39
BMI (kg/m^2^)	24.3 ± 1.5	23.9 ± 0.6	0.75

**Table 2:** Immunological comparison of the non-AD group vs. the AD group.

Parameter	Non-AD (Mean ± SEM)	AD (Mean ± SEM)	*p*-Value
Lymphocytes (%)	15.01 ± 2.33	13.48 ± 2.25	0.64
Monocytes (%)	6.93 ± 0.40	10.31 ± 0.93	0.0029
Neutrophils (%)	76.91 ± 2.69	76.21 ± 2.88	0.86
Eosinophils (%)	0.79 ± 0.22	0.25 ± 0.14	0.046
Basophils (%)	0.36 ± 0.04	0.23 ± 0.04	0.020

**Table 3:** Simple linear regression of p-tau217 and platelet parameters.

Predictor	β (Slope)	95% CI (β)	R^2^	*p*-Value
THR	−0.0193	−0.0373 to −0.0013	0.105	0.036
MPV	1.851	0.237 to 3.465	0.118	0.026

**Table 4:** Simple linear regression of p-tau217 with inflammatory and hepatic biomarkers.

Predictor	β (Slope)	95% CI (β)	R^2^	*p*-Value
Ferritin	0.00538	0.00228–0.00848	0.26	0.0012
LDH	0.0316	0.0178–0.0453	0.35	<0.0001
GOT	0.124	0.0456–0.203	0.21	0.0028

**Table 5:** A logistic regression analysis of SDC3 as a predictor of AD.

Predictor	β (SE)	*p*-Value	OR (Exp(β))	95% CI (OR)	AUC	AUC CI (95%)
SDC3	0.0052 (0.0025)	0.041	1.005	1.001–1.011	0.682	0.519–0.844

Logistic regression was performed with AD diagnosis as the dependent variable (1 = AD, 0 = non-AD) and SDC3 expression as the sole predictor. OR: Odds Ratio; AUC: Area Under the Curve (ROC).

**Table 6:** SDC3 expression in PBMCs by sex and diagnostic group.

	Females (Mean ± SEM)	Males (Mean ± SEM)	*p*-Value
non-AD	147.26 ± 31.46	60.13 ± 22.96	0.058
AD	250.99 ± 49.17	124.55 ± 38.19	0.15
*p*-value	0.11	0.15	

Values represent mean ± SEM. Comparisons were made using unpaired two-tailed *t*-tests within and between groups. No comparisons reached statistical significance, though a trend toward higher SDC3 levels in women was observed in both diagnostic categories.

**Table 7:** Blood pressure comparison between hypertensive and normotensive groups.

Group	Systolic BP (mmHg)	Diastolic BP (mmHg)
HT Patients	127.1 ± 28.7	73.8 ± 15.0
Normotensive	127.7 ± 18.0	76.4 ± 12.3
*p*-value	0.95	0.56

## Data Availability

Data are contained within the article or Supplementary Materials.

## Supplementary Materials

The following supporting information can be downloaded at https://www.mdpi.com/article/10.3390/ijms26146587/s1.
