# Supplementary material for: Peripheral Leukocyte Syndecan-3 Is Elevated in Alzheimer’s Disease: Evidence from a Human Study
Source: Int J Mol Sci. 2025 Jul 9;26(14):6587. doi: 10.3390/ijms26146587 (PMC12294132; doi:10.3390/ijms26146587)
Supplement: Supplementary file 1 [file ijms-26-06587-s001.zip › ijms-3703401-supplementary.pdf]

Supplementary Materials

Peripheral Leukocyte Syndecan-3 Is Elevated in Alzheimer's Disease: Evidence from a Human Study

Anett Hudák, Annamária Letoha, and Tamás Letoha

Supplementary Table S1. Prevalence of Comorbid Conditions in non-AD and AD Groups

Comorbidity	non-AD Group (n = 20)	AD Group (n = 22)
Hypertension	10 (50.0%)	13 (59.1%)
Cardiovascular disease	9 (45.0%)	16 (72.7%)
Diabetes mellitus (any)	4 (20.0%)	4 (18.2%)
Insulin therapy (IDDM)	2 (10.0%)	2 (9.1%)
Chronic lung disease	2 (10.0%)	3 (13.6%)
Renal insufficiency	1 (5.0%)	1 (4.5%)
Autoimmune disorders	2 (10.0%)	3 (13.6%)
Obesity	3 (15.0%)	3 (13.6%)
Depression	1 (5.0%)	4 (18.2%)
Neuropsychiatric (other)	0	2 (schizophrenia, Parkinson's)
Thyroid disorders	0	3 (13.6%)
Gynecological conditions	4 (20.0%)	1 (4.5%)

The table summarizes the frequency and percentage of common comorbidities in the non-AD (n =20) and the AD group (n = 22), including cardiovascular, metabolic, autoimmune, pulmonary, and neuropsychiatric conditions. Comorbidities were identified based on clinical history, medication records, and physician diagnosis. "Neuropsychiatric (other)" includes major neurological or psychiatric disorders other than Alzheimer's disease, specifically schizophrenia and Parkinson's disease. "Insulin therapy" refers to individuals receiving any form of insulin treatment (IDDM). Gynecological conditions were only assessed in female participants.

Supplementary Table S2. Commonly Used Medications in non-AD and AD Groups by Category

Drug Category	non-AD	AD
Antihypertensives	Perindopril, amlodipine, nebivolol	Perindopril, telmisartan, amlodipine, carvedilol
Gastrointestinal protection	Pantoprazole	Pantoprazole, famotidine
Psychotropics	Alprazolam	Escitalopram, risperidone, valproate, alprazolam
Antidiabetics	Metformin, insulin	Insulin, metformin, dapagliflozin
Cardiovascular (platelets, anticoagulants)	Aspirin, clopidogrel, statins	Aspirin, clopidogrel, apixaban, rivaroxaban
Cognitive enhancers	—	Donepezil, memantine, piracetam, vinpocetine
Neuroprotective supplements	Vitamin D, B-complex	Vitamin D, B-complex, Ginkgo biloba

The table lists the most frequently prescribed or reported medications in each drug category among participants in the control and AD groups. Medication data were collected from clinical charts, prescription records, and patient interviews. Drug categories reflect therapeutic intent, including cardiovascular risk management, neuropsychiatric symptom control, and cognitive or neuroprotective support. “Cognitive enhancers” were used exclusively in the AD group. “Neuroprotective supplements” include over-the-counter products with purported cognitive or vascular benefits.

Supplementary Table S3. SDC3 and p-tau217 ELISA Measurements Across AD and Non-AD Subjects

Group	SDC3 value 1 (pg/mL)	SDC3 value 2 (pg/mL)	Mean	% Difference	p-tau217 value 1 (pg/mL)	p-tau217 value 2 (pg/mL)	Mean	% Difference
AD	180.29	196.68	188.49	8.69%	3.02	3.30	3.16	9.00%
AD	15.43	17.79	16.61	14.24%	0.62	0.74	0.68	16.65%
AD	239.89	256.99	248.44	6.88%	2.65	2.48	2.57	6.66%
AD	265.51	246.31	255.91	7.50%	2.56	2.69	2.62	4.81%
AD	137.16	147.19	142.18	7.05%	2.83	2.72	2.78	4.12%
AD	288.81	329.76	309.28	13.24%	5.23	4.26	4.74	20.47%
AD	70.29	62.22	66.26	12.19%	2.56	2.93	2.75	13.59%
AD	22.52	18.98	20.75	17.07%	14.94	12.05	13.50	21.42%
AD	0.00	0.00	0.00	0.00%	1.36	1.17	1.27	15.58%
AD	508.16	500.97	504.57	1.42%	3.14	3.02	3.08	3.97%
AD	334.98	304.61	319.79	9.50%	0.81	0.90	0.86	9.89%
AD	276.12	255.93	266.02	7.59%	9.45	9.97	9.71	5.34%
AD	641.44	633.09	637.27	1.31%	1.75	1.65	1.70	5.61%
AD	549.25	504.05	526.65	8.58%	1.34	1.09	1.22	20.51%
AD	295.14	278.24	286.69	5.90%	4.89	3.94	4.42	21.48%
AD	80.63	72.60	76.61	10.49%	9.52	8.98	9.25	5.81%
AD	7.12	8.31	7.72	15.40%	0.96	0.84	0.90	12.35%
AD	227.00	196.68	211.84	14.31%	1.80	1.45	1.62	21.35%
AD	27.23	28.41	27.82	4.23%	1.86	1.76	1.81	5.49%
AD	379.64	365.14	372.39	3.89%	8.74	7.46	8.10	15.81%
AD	37.79	44.80	41.30	16.97%	6.36	7.09	6.73	10.81%
AD	260.19	212.96	236.58	19.96%	1.18	0.98	1.08	18.15%
Non-AD	128.22	143.85	136.03	11.49%	1.21	1.37	1.29	12.36%
Non-AD	3.55	4.12	3.84	14.81%	6.06	5.43	5.74	10.96%
Non-AD	95.47	107.95	101.71	12.27%	0.50	0.52	0.51	2.18%
Non-AD	66.84	58.75	62.79	12.88%	3.94	4.52	4.23	13.70%
Non-AD	0.00	0.00	0.00	0.00%	9.52	9.97	9.74	4.57%
Non-AD	264.44	272.94	268.69	3.16%	2.90	2.35	2.62	20.99%
Non-AD	24.88	20.16	22.52	20.95%	3.64	4.26	3.95	15.59%
Non-AD	301.46	352.68	327.07	15.66%	8.70	7.46	8.08	15.42%
Non-AD	15.43	17.28	16.35	11.32%	1.78	1.96	1.87	9.48%
Non-AD	0.00	0.00	0.00	0.00%	0.75	0.62	0.69	18.53%
Non-AD	159.38	137.16	148.27	14.98%	2.35	1.99	2.17	16.69%
Non-AD	87.50	102.29	94.89	15.59%	0.11	0.12	0.11	7.99%
Non-AD	57.59	47.13	52.36	19.97%	1.22	1.24	1.23	1.57%
Non-AD	172.61	181.39	177.00	4.96%	1.95	1.76	1.85	10.19%
Non-AD	227.00	235.60	231.30	3.72%	2.97	2.80	2.88	5.81%
Non-AD	13.06	10.78	11.92	19.11%	1.88	2.01	1.95	6.68%
Non-AD	208.63	222.69	215.66	6.52%	0.35	0.41	0.38	14.67%
Non-AD	3.55	2.93	3.24	19.23%	37.84	32.15	35.00	16.25%
Non-AD	217.29	206.46	211.88	5.11%	0.96	1.04	1.00	8.50%
Non-AD	178.10	147.19	162.64	19.01%	3.25	3.05	3.15	6.20%

SDC3 and p-tau217 levels were measured in peripheral blood samples using validated ELISA kits in technical duplicates. The Mean represents the average of duplicate measurements. No evidence of assay saturation or technical anomalies was observed.
